
JOURNAL INFORMATION
==============================
NLM Title Abbreviation: Wirtschaftsdienst
Journal ID: Wirtschaftsdienst
NLM Journal ID: 101086612

Wirtschaftsdienst (Hamburg, Germany : 1949)

ISSN: 0043-6275

ARTICLE INFORMATION
==============================
PMCID: PMC7335919
PMID: 32834152
DOI: 10.1007/s10273-020-2668-5
Article ID: 2668
Article version: 1
Subjects: Zeitgespräch

Die europäische Antwort auf die Corona-Pandemie

Südekum Jens suedekum@dice.hhu.de

grid.411327.2 0000 0001 2176 9917 Düsseldorfer Institut für Wettbewerbsökonomie, Heinrich-Heine-Universität Düsseldorf, Universitätsstraße 1, 40225 Düsseldorf, Deutschland
Electronic publication date: 2020 Jul 6
Print publication date: 2020
Volume: 100
Issue: 6
First page: 397
Last page: 400
Copyright: © Der/die Autor(en) 2020
License: Open Access Dieser Artikel wird unter der Creative Commons Namensnennung 4.0 International Lizenz veröffentlicht, welche die Nutzung, Vervielfältigung, Bearbeitung, Verbreitung und Wiedergabe in jeglichem Medium und Format erlaubt, sofern Sie den/die ursprünglichen Autor(en) und die Quelle ordnungsgemäß nennen, einen Link zur Creative Commons Lizenz beifügen und angeben, ob Änderungen vorgenommen wurden.
Die in diesem Artikel enthaltenen Bilder und sonstiges Drittmaterial unterliegen ebenfalls der genannten Creative Commons Lizenz, sofern sich aus der Abbildungslegende nichts anderes ergibt. Sofern das betreffende Material nicht unter der genannten Creative Commons Lizenz steht und die betreffende Handlung nicht nach gesetzlichen Vorschriften erlaubt ist, ist für die oben aufgeführten Weiterverwendungen des Materials die Einwilligung des jeweiligen Rechteinhabers einzuholen.
Weitere Details zur Lizenz entnehmen Sie bitte der Lizenzinformation auf http://creativecommons.org/licenses/by/4.0/deed.de.
License URL: https://creativecommons.org/licenses/by/4.0/

issue-copyright-statement: © The Author(s) 2020

==============================

Um die wirtschaftlichen Schäden der Corona-Pandemie abzufedern, wurde in Deutschland ein umfangreicher Rettungsschirm aufgespannt. Der Bundestag verabschiedete einen Nachtragshaushalt über 156 Mrd. Euro, die Länderhaushalte zogen nach. Millionen Beschäftigte nehmen Kurzarbeit in Anspruch. Es wurden umfangreiche Steuerstundungen gewährt und zusätzlich Garantien vergeben, damit sich die Unternehmen praktisch unbegrenzt mit staatlich garantierten Liquiditätskrediten versorgen können. Für Kleinstbetriebe und Solo-Selbständige gab es finanzielle Soforthilfen, für Großunternehmen den Wirtschaftsstabilisierungsfonds (WSF).

Abbildung 1 zeigt, dass sich die Maßnahmen auf insgesamt 1,8 Billionen Euro summieren. Das entspricht rund 50 % des Bruttoinlandsprodukts (BIP), die mobilisiert werden könnten – auch wenn die tatsächliche Inanspruchnahme der Kreditlinien weitaus geringer ausfällt. Damit gehört das deutsche Rettungsprogramm zu den großzügigsten weltweit. Dessen Umfang könnte aber auch noch zunehmen. So ist am 3. Juni 2020 ein groß angelegtes Konjunkturprogramm im Umfang von 130 Mrd. Euro angekündigt worden. Und sollte es nach den aktuellen Lockerungen zu einer zweiten Welle der Virusverbreitung kommen, die wiederum breit angelegte Eindämmungsmaßnahmen erforderlich machen, dann wird es noch weitaus teurer werden.

Glücklicherweise ist Deutschland in der Lage, mit erheblicher fiskalischer Feuerkraft gegen die Corona-Krise anzusteuern. Finanzierungsschwierigkeiten sind nirgends zu erkennen. Im Gegenteil, der Zinssatz für die zehnjährige Bundesanleihe sank von Ende Januar 2020 bis Ende Mai sogar von -0,2 % auf unter -0,4 %. Dies hängt auch damit zusammen, dass weltweit Kapital aus Entwicklungs- und Schwellenländern in die sicheren Häfen abfließt, wozu Deutschland gehört.

In anderen Ländern der Europäischen Union ist die Situation jedoch weit weniger komfortabel. Italien wurde als erstes Land in Europa vom Corona-Virus getroffen. Es hat heute, bezogen auf die Bevölkerungsgröße, rund doppelt so viele Fälle und fünfmal mehr Tote als Deutschland (Worldometer, 2020), aber weniger als ein Drittel der intensivmedizinischen Kapazitäten (Science Media Center, 2020). In Spanien sieht es ähnlich verheerend aus. Die Wirtschaft wurde in beiden Ländern mit wenigen Ausnahmen, anders als in Deutschland, komplett heruntergefahren. Hinzu kamen härtere Ausgangssperren als hierzulande. Damit sind auch die zu befürchtenden wirtschaftlichen Folgeschäden noch weitaus schlimmer.

Eigentlich müssten Italien und Spanien deshalb noch schwerere Geschütze als Deutschland auffahren, um die Krise zu bekämpfen. Doch das Gegenteil ist der Fall. Zwar wurden die Maastrichter Schuldenregeln von der EU-Kommission zügig außer Kraft gesetzt. Aber die fiskalischen Gegenmaßnahmen belaufen sich (inklusive aller staatlichen Garantien) in Spanien nur auf etwa 11 % des BIP (Bruegel, 2020). Der direkte fiskalische Impuls ist in Italien mit lediglich 0,9 % des BIP ebenfalls gering. Allerdings wurden über den staatlichen Exportversicherer SACE vergleichsweise großzügige Garantien für Liquiditätskredite mobilisiert (vgl. Abbildung 1).

Dafür haben sich die italienischen Finanzierungskonditionen seit Ende Januar bereits spürbar verschlechtert, von 0,92 % für die zehnjährige Anleihe auf aktuell 1,53 %. Am 18. März 2020 drohte kurzzeitig sogar der komplette Verlust des Marktzugangs für Italien und der Zinsaufschlag stieg sprunghaft auf über 300 Basispunkte an, ehe die Europäische Zentralbank die Märkte durch die Einführung des Pandemic Emergency Purchase Programme (PEPP) mit einem Gesamtvolumen von zunächst 750 Mrd. Euro wieder beruhigen konnte.

Diese Schwierigkeiten spiegeln auch den Verschuldungsgrad beider Länder wider. Zwar hat Italien in den vergangenen 25 Jahren praktisch ununterbrochen Haushalte mit Primärüberschüssen vorgelegt, aber aufgrund niedrigen Wachstums und einer weiterhin ungelösten Altschuldenproblematik aus den 1980er Jahren steht das Land mit einer Schuldenquote von 134 % des BIP in Europa so schlecht da wie sonst nur Griechenland. Spanien war einmal ein Mus-

Abbildung 1
**Diskretionäre fiskalpolitische Maßnahmen gegen die Corona-Pandemie**
in % des Bruttoinlandsprodukts, 2019

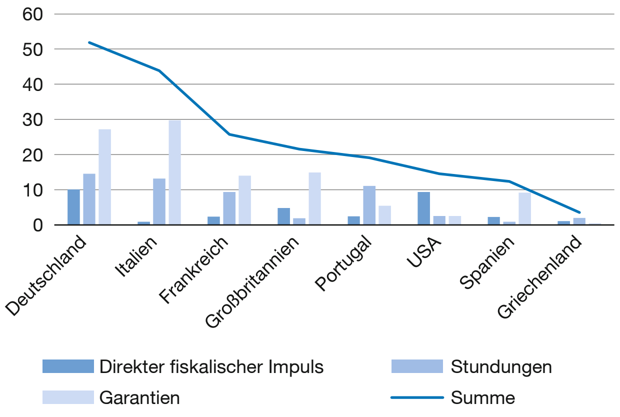

Quelle: Bruegel (2020).

terschüler mit nur 35 % Staatsschuldenquote im Jahr 2007. Im Zuge der Finanzkrise ist die Quote aber auf 100 % gewachsen. Dies erweist sich heute als Hemmschuh für eine fiskalische Reaktion.

## Symmetrischer Schock trifft auf asymmetrische Bedingungen

Bei der Corona-Pandemie handelt es sich nicht um einen asymmetrischen Schock, der seinen Ursprung in wirtschaftspolitischen Fehlern hatte. Corona trifft ganz Europa symmetrisch. Rein zufällig kamen einige Länder durch Dienstreisen oder Tourismus früher mit dem Virus in Kontakt. Andere waren erst später dran und konnten sich besser vorbereiten. Mit Schuld hat das nichts zu tun. Italien und Spanien hatten einfach Pech, Griechenland Glück, denn dort sind die Corona-Fallzahlen relativ klein.

Der symmetrische Schock trifft aber auf höchst asymmetrische Kapazitäten für ein gesundheits-, wirtschafts- und finanzpolitisches Gegensteuern. Dies kann die Funktionsfähigkeit des europäischen Binnenmarkts ernsthaft beschädigen und im Extremfall die gesamte Europäische Union vor eine Zerreißprobe stellen, wenn lediglich einige finanzstarke Mitgliedstaaten große Rettungsschirme für ihre Unternehmen und deren Beschäftigte aufspannen können, während andere Länder (hauptsächlich aufgrund ihrer finanzpolitischen Vergangenheit) nicht im selben Ausmaß dazu in der Lage sind.

Das europäische Beihilferecht ist derzeit durch die Annahme eines Sonderrahmens recht großzügig, weil staatliche Hilfspakete notwendig und erwünscht sind. Aber eine rein nationalstaatliche Ausgestaltung der Rettungspolitik kann schnell zu Marktverschiebungen innerhalb der EU führen und damit gleichermaßen die Ziele von unverzerrtem Wettbewerb und Konvergenz verletzen.

## Erste Phase der europäischen Rettungspolitik: Versicherung

Weil die Corona-Pandemie ein symmetrischer, gesamteuropäischer Schock ist, sollten es die Gegenmaßnahmen aus ordnungspolitischer Sicht daher auch sein. Europa bräuchte ein gemeinsames Paket, das für alle zugänglich ist und Geld dorthin verteilt, wo die wirtschaftlichen Schäden am schlimmsten sind.

Am 9. April 2020 verabschiedeten die Staats- und Regierungschefs der EU ein erstes gesamteuropäisches Maßnahmenpaket, das aus drei wesentlichen Säulen besteht:

1. Einer Pandemie-Krisenhilfe (Pandemic Crisis Support Instrument, PCSI) des Europäischen Stabilitätsmechanismus (ESM). Hiernach können die Mitgliedstaaten Kredite im Umfang von bis zu 2 % des BIP aus der vorsorglichen ESM-Kreditlinie abrufen. Die einzige Bedingung besteht darin, dass die Mittel zur Finanzierung gesundheitspolitischer Maßnahmen bzw. zur Bekämpfung der Corona-Pandemie eingesetzt werden.

2. Einem Garantiefonds der Europäischen Investitionsbank (EIB), der von den Mitgliedstaaten mit Haushaltsgarantien im Umfang von 25 Mrd. Euro ausgestattet wurde. Damit sollen Liquiditätskredite an kleine und mittelständische Unternehmen (KMU) im Umfang von rund 200 Mrd. Euro abgesichert werden.

3. Dem europäischen SURE (Support Mitigating Unemployment Risks in Emergency)-Programm mit einem Gesamtumfang von 100 Mrd. Euro. In dessen Rahmen werden Kredite an die Mitgliedstaaten vergeben, um Unternehmen eine Weiterbeschäftigung ihrer Mitarbeiter während der Krise zu finanzieren.

Die beiden letzten Säulen replizieren die aus dem deutschen Kontext bekannten Instrumente der Liquiditätskredite der Kreditanstalt für Wiederaufbau (KfW) und des Kurzarbeitergelds. Ihre Konzeption folgt damit einem Versicherungsgedanken: die Verhinderung von Hysteresis-Effekten durch Unternehmensinsolvenzen und Arbeitslosigkeit. Der finanzielle Gesamtumfang der beiden ersten Säulen des EU-Pakets ist aber vergleichsweise überschaubar. Besonders heikel ist die erste Säule. Der ESM ist eine Institution aus der Zeit der Finanz- und Eurokrise. Sein wesentlicher Zweck besteht darin, im Fall von schweren asymmetrischen Schocks Euro-Ländern durch Kreditzusagen den Zugang zum Kapitalmarkt zu gewährleisten. Dafür werden üblicherweise strikte Reformauflagen eingefordert.

In der Corona-Krise wurde auf solche strikte Konditionalität verzichtet. Dennoch haftet den ESM-Krediten ein gewisses Stigma an. Es war daher nicht verwunderlich, dass sich Italien gegen die ESM-Lösung ausgesprochen und die entsprechenden PCSI (Pandemic Crisis Support-Instrument)-Kreditlinien bislang nicht in Anspruch genommen hat. Von einigen deutschen Kommentatoren wurde das so gewertet, dass das Land offenbar keinen akuten Finanzierungsbedarf habe.[1] Diese Interpretation ist aber nicht zwangsläufig, da allein die Existenz einer ESM-Kreditlinie (auch ohne Beanspruchung) ein entsprechendes Signal an die Märkte darstellt. Hierdurch werden die Finanzierungskonditionen italienischer Staatsanleihen verbessert, zumal auch das PEPP der Europäischen Zentralbank einen entsprechenden Beitrag leistet. In der Folge konnten die italienischen und spanischen Zinsaufschläge bislang stabil gehalten werden.

Dies kann sich aber schnell ändern, sobald die Märkte die Funktionalität des PEPP – oder die mögliche alternative Absicherung des Marktzugangs über das bereits existierende Outright-Monetary-Transactions-(OMT)-Programm – in Zweifel ziehen. Erneute Klagen gegen die EZB-Programme vor nationalen Gerichten (mutmaßlich dem deutschen Bundesverfassungsgericht) könnten diese Zweifel weiter nähren, weil eine zu zögerliche Reaktion der EZB aufgrund der juristischen Unwägbarkeiten antizipiert werden könnte. In diesem Fall drohte die Rückkehr der Eurokrise.

Die in Abbildung 1 aufgezeigten relativ geringen direkten fiskalischen Impulse in den stark von Corona betroffenen südeuropäischen Volkswirtschaften dürften auch vor diesem Hintergrund zu sehen sein: Unsicherheit über den Fortbestand der derzeit noch günstigen Refinanzierungsbedingungen, die stark von exogenen Faktoren (wie dem Funktionieren bestimmter EZB-Programme) abhängen, die von den Ländern nicht direkt beeinflussbar sind.

## Zweite Phase der europäischen Rettungspolitik: Wiederaufbau

Nach den unmittelbaren Versicherungsmaßnahmen der ersten Phase steht als zweiter Schritt nun ein Konjunkturprogramm auf der Agenda. Das Ziel dieser zweiten Phase, die oft als „Wiederaufbau“ bezeichnet wird, liegt vor allem in der Verbreiterung konjunktureller Nachholeffekte. Teilweise ergeben sich diese von selber: Aufgeschobene Käufe

1 So sagte Friedrich Merz am 19.4.2020, Italien „versuche im Windschatten von Corona zu unbegrenzten Refinanzierungsmöglichkeiten für seinen Staatshaushalt zu kommen“ (Zeit-online, 2020).

Abbildung 2

**Vorschlag der EU-Kommission zur Aufteilung der Mittel aus dem European Recovery Fund**

in Mrd. Euro

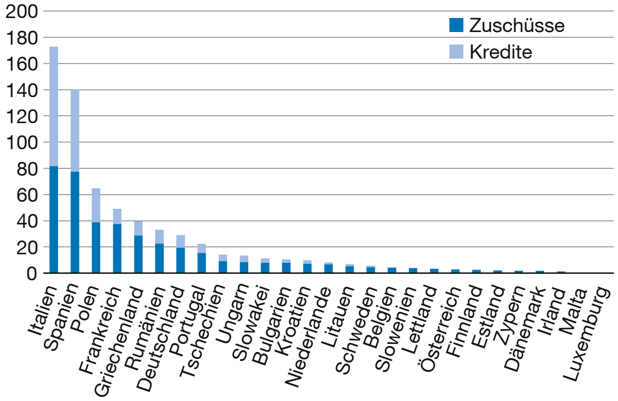

Quelle: Kafsack (2020).

langlebiger Konsumgüter können jetzt nachgeholt werden. In anderen Branchen (wie dem Tourismus oder bei Veranstaltungen) ist dies aber weiterhin nur eingeschränkt möglich, sodass für einen konjunkturellen Aufschwung anderer Konsum an diese Stelle treten muss. Zudem herrscht bei etlichen Beschäftigten und Unternehmen weiterhin eine Konsum- bzw. Investitionszurückhaltung vor, sei es aufgrund von Schuldenüberhang durch Liquiditätskredite, oder aus genereller Sorge vor der weiteren Pandemieentwicklung und ihren wirtschaftlichen Folgen. Hier kann ein staatliches Konjunkturprogramm eine entsprechende positive Erwartungsbildung schaffen und somit private Nachfrage ankurbeln.

Würde diese zweite Phase wiederum vor allem nationalstaatlich organisiert, ergäben sich dieselben Probleme wie schon in der ersten Phase. Es drohte ein weiteres Auseinanderdriften der Europäischen Union, wenn finanzstärkere Mitgliedsländer größere Pakete auf den Weg brächten. Es ist daher folgerichtig, dass auch in dieser Phase eine gesamteuropäische Antwort angestrebt wird.

Den Anfang machte ein deutsch-französischer Vorschlag, der kurz darauf von der EU-Kommission aufgegriffen und erweitert wurde. Dessen Grundlage ist ein „European Recovery Fund“ neben dem regulären EU-Budget, finanziert durch die Ausgabe gesamteuropäischer Anleihen im Umfang von 750 Mrd. Euro. Diese EU-Anleihen sind nicht wie klassische Eurobonds in gesamtschuldnerischer Haftung konzipiert, sondern die Mitgliedstaaten haften lediglich mit ihren Beiträgen zum EU-Budget. Die Mittel sollen zu einem Drittel als Kredite und zu zwei Drittel als Zuschüsse an die Mitgliedstaaten vergeben werden, um damit konjunkturwirksame und gleichsam strukturbildende Investitionen zu finanzieren. Da der Vorschlag den Eigenmittelbeschluss in

Art. 311 des EU-Vertrags berührt, erfordert er Einstimmigkeit aller EU-Mitglieder. Dieser Diskussionsprozess ist derzeit im Gange. Wesentlicher Streitpunkt ist dabei, neben dem Gesamtvolumen, die überwiegende Verausgabung der Mittel in Form von Zuschüssen. Hiergegen haben sich insbesondere die sogenannten „sparsamen Vier“ (Niederlande, Österreich, Dänemark und Schweden) in Stellung gebracht, die eine Ausgestaltung ausschließlich in Form von Krediten bevorzugen.

Die Zuweisung der Mittel auf die einzelnen Mitgliedstaaten ist ebenfalls noch Teil der laufenden Verhandlungen. Die in Abbildung 2 ursprünglich von der EU-Kommission angedachte Verteilung kann sich deshalb auch noch ändern. Viele weitere Aspekte, wie die inhaltlichen Schwerpunkte der Investitionsprogramme, die Finanzierungsstruktur der Anleihen, Fragen der nationalen Ko-Finanzierung etc. sind auch noch offen. Aus ökonomischer Sicht sollten die angestrebten Fristigkeiten möglichst langfristig sein. Wenn die EU-Anleihe einen ähnlichen Bonitätsstatus wie die deutsche Bundesanleihe (AAA) erreicht, dann dürften die Nominalzinsen selbst bei Laufzeiten von 30 Jahren in der Nähe von null liegen, real also deutlich im negativen Bereich.[2] Hierzu trägt auch bei, dass die EZB die EU-Anleihen im Rahmen ihrer Kaufprogramme als Sicherheiten akzeptiert.

Die sich daraus ergebenden Finanzierungsspielräume dürfen nicht leichtfertig verspielt werden. Es wäre nicht sachgerecht, auf kurze Laufzeiten zu setzen, um Tilgungen zu beschleunigen. Denn diese konterkarieren den angestrebten Konjunktur- und Wachstumsimpuls ja geradewegs wieder. Europa hat stattdessen die Möglichkeit, aus den Schulden wieder herauszuwachsen, die Schuldenquote im Zeitablauf also wieder abzusenken, ohne im buchstäblichen Sinne zu tilgen. Die vorwiegende Vergabe von Zuschüssen sollte auch erhalten bleiben. Denn eine weitere Vergabe von Krediten an die ohnehin hoch verschuldeten Mitgliedstaaten wird der Natur der Corona-Krise nicht gerecht: ein symmetrischer Schock bei asymmetrischer Reaktionsfähigkeit.

Schließlich wäre es wünschenswert, wenn durch Corona ein grundsätzlicher Diskussionsprozess über die Architektur der EU und der Eurozone in Gang käme. Nach 13 Jahren Krise sollte mittlerweile deutlich geworden sein, dass die ursprüngliche Konzeption des Euro nicht kompatibel mit der Realität ist. Eine gemeinsame Währung benötigt zur Stabilisierung eine zentralisierte Fiskalkapazität und eine verlässlich akkomodierende Geldpolitik – ohne permanente juristische Imponderabilien. Die EU-Verträge sehen diese Elemente nicht wirklich vor. Darüber wird in Zukunft zu sprechen sein.

2 Es waren schon seit längerem EU-Anleihen im Umfang von rund 30 Mrd. Euro im Umlauf. Auch das SURE-Programm wird über derartige Anleihen finanziert, deren Rating typischerweise das Niveau AAA erreicht (European Commission, 2020).

Prof. Dr. Jens Südekum ist Professor am Düsseldorf Institute for Competition Economics (DICE) der Heinrich-Heine-Universität Düsseldorf.


Literatur

Bruegel (2020), The fiscal response to the economic fallout from the coronavirus, Bruegel, https://www.bruegel.org/publications/datasets/covid-national-dataset (25. Mai 2020).
European Commission (2020), Investor Presentation, May 2020, https://ec.europa.eu/info/sites/info/files/economy-finance/eujnvestor_presentation_en.pdf (4. Juni 2020).
Kafsack, H. (2020), So viel Geld erhalten die einzelnen Staaten, Frankfurter Allgemeine Zeitung, 27. Mai, https://www.faz.net/aktuell/wirtschaft/750-milliarden-euro-der-eu-so-viel-erhalten-die-einzelnen-staaten-16788431.html (27. Mai 2020).
Science Media Center (2020), Auslastung der Intensivstationen: Zahlen aus Deutschland und Europa, https://www.sciencemediacenter.de/alle-angebote/fact-sheet/details/news/auslastung-der-intensivstati-onen-zahlen-aus-deutschland-und-europa/ (4. Juni 2020).
Worldometer (2020), COVID-19 Coronavirus Pandemic, https://www.worldometers.info/coronavirus/ (2. Mai 2020).
Zeit-online (2020), Friedrich Merz fordert mehr Geld für EU, https://www.zeit.de/politik/2020-04/europaeische-union-friedrich-merz-cdu-geld-ratspraesidentschaft (4. Juni 2020).
